# Inhibition of GLS suppresses proliferation and promotes apoptosis in prostate cancer

**DOI:** 10.1042/BSR20181826

**Published:** 2019-06-25

**Authors:** Junfeng Zhang, Shiyu Mao, Yadong Guo, Yuan Wu, Xudong Yao, Yong Huang

**Affiliations:** 1 Department of Urology, Shanghai Tenth People’s Hospital, Tongji University School of Medicine, Shanghai, China; 2 Department of Urology, The Affiliated Hospital of Inner Mongolia Medical University, Huhhot, China

**Keywords:** apoptosis, GLS, proliferation, prostate cancer

## Abstract

Altered glutamine metabolism is a hallmark of cancer growth, forming the theoretical basis for development of metabolic therapies as cancer treatments. Glutaminase (GLS), a crucial enzyme involved in the regulation of glutamine metabolism, has been reported to play crucial roles in cancer development. However, the precise function of GLS in prostate cancer (PCa) remains unclear. The purpose of the present study was to assess the GLS expression and its clinical significance in PCa. We found that GLS was significantly up-regulated in PCa tissues and cell lines. High expression of GLS was significantly associated with Gleason score (*P*=0.001) and Tumor stage (*P*=0.015). Functionally, we silenced GLS in PCa cell lines and revealed that GLS knockdown largely blunted the proliferation of DU145 and PC-3 cells. Mechanistically, we demonstrated that knockdown of GLS induced apoptosis and cell cycle arrest. Moreover, we observed that the expressions of Bax were increased while the levels of cyclinD1 and Bcl-2 were decreased after knockdown of GLS in PCa cells. Importantly, through Western blot analysis, we identified that GLS knockdown dramatically suppressed Wnt/β-catenin pathway. Taken together, GLS is a novel oncogene in PCa and may be a potential treatment target for PCa patients.

## Introduction

Prostate cancer (PCa) is one of the most common malignancy among men and is the second major cause of male cancer-related deaths in the United States [1]. In 2016, the American Cancer Society reported that approximately 180890 new PCa cases and 26120 deaths occurred in United States [2]. Although most PCa cases are treatable among men diagnosed with localized or regional disease as evidenced by a 100% 5-year survival rate. However, the 5-year survival rate drops to 29% for patients who develop metastatic disease [3]. Therefore, a more thorough understanding of the mechanisms underlying PCa pathogenesis is urgently needed for early diagnosis and treatment of PCa patients.

Glutaminase (GLS), which converts glutamine into glutamate, plays a vital role in up-regulating cell metabolism for tumor cell growth [4]. Recent study found that the expression of GLS is up-regulated and correlates with pathological factors in leukemia [5], glioma [6], melanoma [7], as well as in pancreas [8], bladder [9], lung [10], and breast cancers [11]. Many oncogenes and tumor suppressors have been linked to the regulation of GLS expression and glutamine metabolism [12]. For example, c-MYC stimulates glutamine catabolism to fuel growth and proliferation of cancer cells through up-regulating GLS [13]. GLS up-regulates glucose uptake through targeting TXNIP in PCa [14]. A recent study suggested an important role of GLS in large extracellular vesicles formation in metastatic PCa [15]. However, its precise mechanism involved in the carcinogenesis of PCa is yet to be elucidated.

In the present study, we investigated the expression and biological roles of GLS in PCa. We found that GLS is overexpressed in PCa tissues and cell lines compared with the adjacent normal tissues and the normal prostate epithelial cell line RWPE-1. In addition, we explored the relationship between GLS expression and the clinicopathological features in PCa patients. Furthermore, we demonstrated that knockdown of GLS significantly inhibited proliferation and induced apoptosis and cell cycle arrest in PCa cell lines DU-145 and PC-3. We observed that knockdown of GLS altered multiple apoptosis and cell cycle related proteins expression in PCa cells. Together, these observations suggest that GLS is up-regulated and may act as an oncogene in PCa.

## Materials and methods

### Tissues’ samples

Sixty-eight paired PCa tissues (T) and the matched normal prostate tissues (N) were obtained from the Shanghai Tenth People’s Hospital, Tongji University School of Medicine (China). These patients did not receive any local or systemic treatment before operation. All specimens had confirmed pathological diagnosis and were classified according to the WHO criteria. The clinicopathological patient information was collected and summarized in Table 1. Our work has been carried out in accordance with The Code of Ethics of the World Medical Association (Declaration of Helsinki). The study was approved by Shanghai Tenth People’s Hospital Ethics Committee and written informed consents were obtained from all patients.

**Table 1:** Correlation of GLS expression with clinicopathological factors in 68 PCa patients

Clinicopathological factor	All cases (*n*=68)	High expression (*n*=34)	Low expression (*n*=34)	*P*-value
Age (years)				0.209
≥65	43	24	19	
<65	25	10	15	
PSA (ng/ml)				0.324
≥10	40	18	22	
<10	28	16	12	
Gleason score				0.001
≥8	43	28	15	
<8	25	6	19	
Tumor stage				0.015
<T3	30	10	20	
≥T3	38	24	14	
Lymphatic invasion				0.380
Presence	15	6	9	
Absence	53	28	25	
Seminal vesicle invasion				0.462
Presence	29	16	13	
Absence	39	18	21	

### Cell culture

PCa cell lines (22Rv1, DU145, PC-3 and LNcaP) and normal prostatic epithelial cell (RWPE-1) were obtained from the American Type Culture Collection (ATCC, Rockville, U.S.A.). DU145, PC-3 and LNcaP were maintained in RPMI-1640 (Gibco; Thermo Fisher Scientific, Inc., Waltham, MA, U.S.A.) supplemented with 10% fetal bovine serum (Gibco), 50 U/ml of penicillin and 50 μg/ml of streptomycin (Invitrogen, U.S.A.). RWPE-1 cells were cultured in keratinocyte serum-free medium supplemented with bovine pituitary extract (0.05 mg/ml) and epidermal growth factor (5 ng/ml). Cells were incubated at 37°C in a humidified atmosphere with 5% CO_2_.

### RNA isolation and quantitative real-time PCR

Total RNA was extracted from human tissues or cultured cells with TRIzol reagent (Invitrogen, CA, U.S.A.), and the corresponding cDNA was generated with the cDNA synthesis kit (Takara, Kyoto, Japan) according to the manufacturer’s instructions. Quantitative real time-PCR (qRT-PCR) was performed using SYBR Green PCR Kit (Takara Biotechnology, Dalian, China) with an ABI Prism 7500 Sequence Detection System (Applied Biosystems, Foster City, CA, U.S.A.). The GLS mRNA level was normalized to the β-Actin mRNA level. Data were analyzed using the 2^−ΔΔ*C*^_t_ method. The primer sequences were as follows: 5′-TTCCAGAAGGCACAGACATGGTTG-3′ (forward) and 5′-GCCAGTGTCGCAGCCATCAC-3′ (reverse) for GLS; 5′-CCTGGCACCCAGCACAAT-3′ (forward) and 5′-GGGCCGGACTCGTCATAC-3′ (reverse) for β-actin.

### Cell transfection

Small interfering RNA specifically targeting human GLS (si-GLS) and scrambled negative control oligos (si-NC) were purchased from Sangon (Shanghai, China). Cell transfections were performed using Lipofectamine 3000 (Invitrogen; Thermo Fisher Scientific, Inc., U.S.A.) according to the manufacturer’s instructions. The siRNA sequences were as follows: si-GLS #1, 5′-GAUGGACAGAGGCAUUCUA-3′ (sense), si-GLS #2, 5′-CCCAGGUUGAAAGAGUGUA -3′ (sense). The siRNA sequence with the maximal interfering effect (si-GLS #1) was selected and used for all the subsequent experiments. Total RNA or protein was extracted after 48 h following transfection.

### Cell proliferation

Cell proliferation was measured using Cell Counting Kit-8 (CCK-8; Dojindo, Kumamoto, Japan). The transfected cells were seeded into 96-well plates at a density of 1000 cells/well. Then, 10 μl CCK-8 solution was added to each plate at selected time points and incubated for 2 h at 37°C. The absorbance was measured at 450 nm with a microplate spectrophotometer (BioTek, Winooski, VT, U.S.A.).

### Colony formation assay

Colony formation assays were performed to evaluate the cell proliferation. Briefly, cells were seeded in six-well plates at approximately 1000 cells/well. After 14-day incubation, the cells were harvested and fixed with 75% ethanol, stained with 0.5% Crystal Violet, and visible colonies were counted.

### Apoptosis assay

Cell apoptosis was measured by flow cytometry using Annexin V-FITC Apoptosis Kit (BD Biosciences, Erembodegem, Belgium) in accordance with the manufacturer’s instructions. The transfected cells were collected by 0.25% trypsin without EDTA. After washing with ice-cold PBS, cells were stained with fluorescein isothiocyanate (FITC) and propidium iodide (PI) in the dark at room temperature for 20 min. Apoptosis rate was detected by using BD FACS Calibur (Beckman Coulter, CA, U.S.A.).

### Cell cycle analysis

Cell cycle distribution was measured by PI staining. After 48 h of transfection, cells were harvested and washed twice with cold PBS. Then, cells were fixed in 70% ethanol at 4°C overnight and washed with cold PBS. Cells were collected and resuspended in PBS containing PI in the dark at 37°C for 20 min. Cell cycle distribution was analyzed by flow cytometry using BD FACS Calibur. Three independent experiments were conducted.

### Western blot

The total proteins of cells were extracted in RIPA buffer (Beyotime, Shanghai, China) supplemented with 1% PMSF and 1% protease inhibitor cocktail (Thermo Scientific 78440). The reaction was incubated on ice for 30 min and centrifuged for 10 min (12000×***g***, 4°C). The supernatant was collected, and the protein concentration estimated using a Pierce BCA protein assay kit (Thermo Scientific, Rockford, IL, U.S.A.). Total proteins were separated by sodium lauryl sulfate/polyacrylamide gels (SDS/PAGE) and transferred on to a nitrocellulose (NC) membrane. Membranes were blocked with 5% non-fat milk in PBS for 1 h at room temperature and then incubated with primary antibody against GLS (ab156876, Abcam, Cambridge, MA, U.S.A.), cyclinD1 (ab134175, Abcam), Bcl-2 (ab32124, Abcam), Bax (ab32503, Abcam), β-catenin (ab32572, Abcam) and phospho-β-catenin (ab11350, Abcam). β-actin (ab8226, Abcam) was used as an internal control. The protein band was visualized using the Odyssey scanner (Li-COR Biosciences, Lincoln, NE, U.S.A.).

### Statistical analysis

Data were analyzed using SPSS 15.0 software (Chicago, IL, U.S.A.). Results are presented as mean ± standard deviation (SD) from at least three independent experiments. The Student’s *t* test was used to assess between-group differences. The association between patients’ characteristics and GLS expression was evaluated by Chi-Square test or Fisher’s exact test. *P*<0.05 were considered to a statistically significant difference.

## Results

### GLS is increased in PCa tissues and cell lines

**Figure 1:**
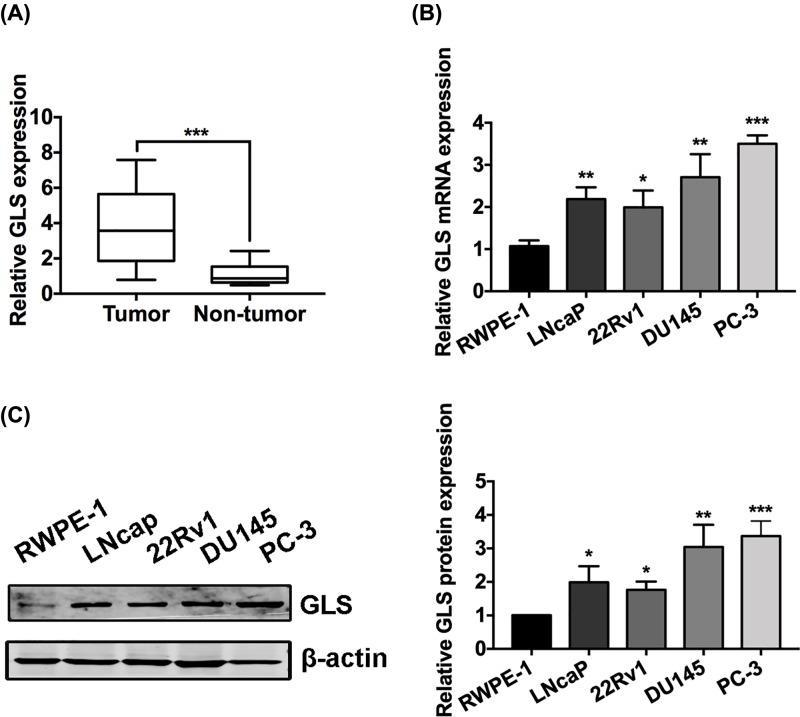
GLS is overexpressed in PCa tissues and cell lines (**A**) Relative mRNA levels of GLS in PCa tissues (Tumor) and normal prostate tissues (non-tumor) detected by qRT-PCR. (**B,C**) Relative mRNA and protein levels of GLS in PCa cell lines (DU145, PC-3 and LNcaP) and normal prostatic epithelial cell line (RWPE-1) detected by qRT-PCR and Western blot, respectively. *P<0.05, **P<0.01, ***P<0.001.

To study the potential roles of GLS in the development of PCa, we detected the expression patterns of GLS in PCa tissues and cell lines. Results of qRT-PCR indicated that GLS is significantly overexpressed in PCa tissues compared with normal prostate tissues (Figure 1A). In addition, data from qRT-PCR and Western blot showed that both the mRNA and protein levels of GLS are higher in PCa cell lines (22Rv1, DU145, PC-3 and LNcaP) compared with normal prostatic epithelial cell line RWPE-1 (Figure 1B,C).

### Elevated GLS expression is related to clinicopathologic characters in PCa

The correlation between GLS levels and clinicopathologic features of PCa is summarized in Table 1. The high expression of GLS was significantly associated with Gleason score (*P*=0.001) and Tumor stage (*P*=0.015). However, there was no significant relationship between GLS expression and variables such as Age (*P*=0.209), PSA level (*P*=0.324), Lymphatic invasion (*P*=0.380), and Seminal vesicle invasion (*P*=0.462).

### GLS knockdown suppresses the proliferation of PCa cells

**Figure 2:**
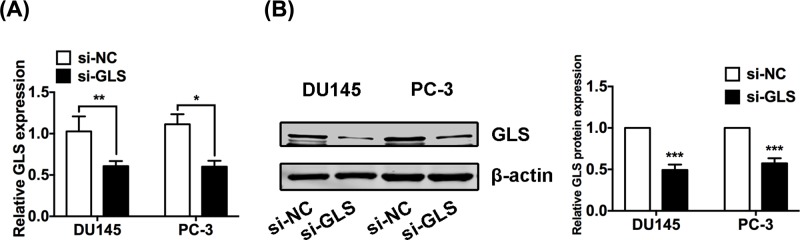
GLS was efficiently silenced in PCa cells (**A,B**) Relative mRNA levels (A) and protein expressions (B) of GLS in PCa cells (DU145 and PC-3) after 48 h transfection of si-NC or si-GLS. *P<0.05, **P<0.01, ***P<0.001.

**Figure 3:**
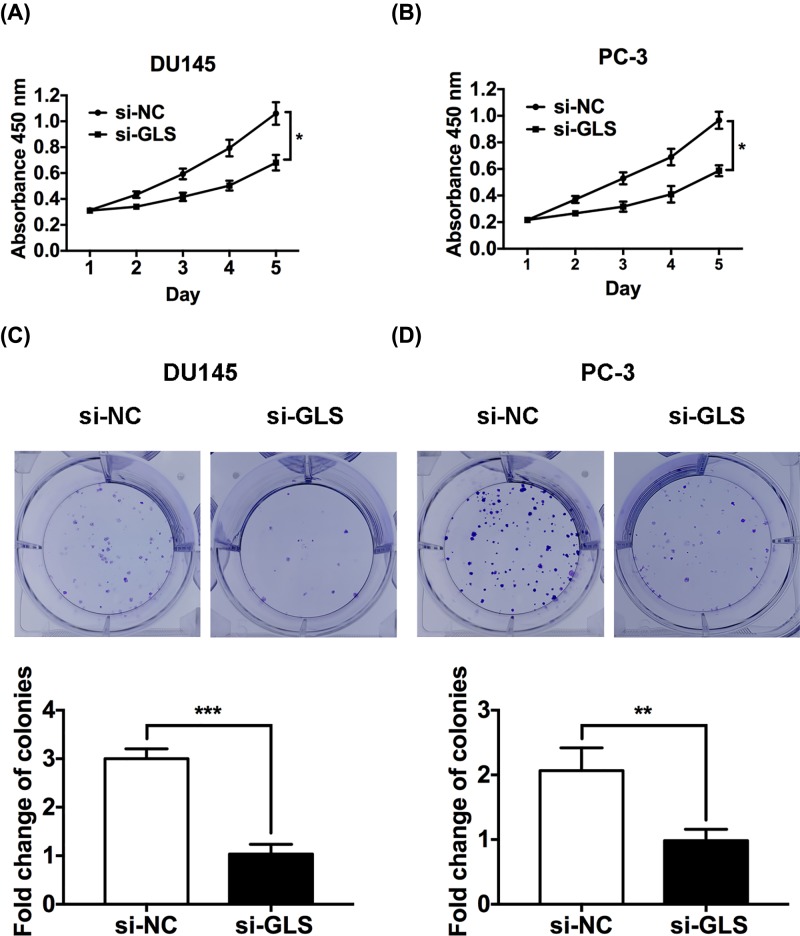
Knockdown of GLS inhibited proliferation in PCa cells (**A,B**) CCK-8 assay revealed cell growth curves of transfected DU145 and PC-3 cells. (**C,D**) Colony formation rates of transfected DU145 and PC-3 cells. *P<0.05, **P<0.01, ***P<0.001.

Small interfering RNA targeting GLS (si-GLS) was transfected to suppress the expression of GLS in PCa cells, and non-specific negative control oligos (si-NC) was used as a control. First, we found that GLS was efficiently silenced in si-GLS DU145 and PC-3 cells as shown by the qRT-PCR and Western blot results (Figure 2). CCK-8 and colony formation assay were performed to investigate cell proliferation after knockdown of GLS. Data of CCK-8 assay showed that knockdown of GLS largely blunted the proliferation of PCa cell lines DU145 and PC-3 (Figure 3A,B). The normal prostatic epithelial cell RWPE-1 was barely affected after knockdown of GLS (Supplementary Figure S1), suggesting that knockdown of GLS can inhibit cancer cells without markedly affecting normal cells. In addition, the colony formation rate of cells was significantly inhibited following GLS knockdown (Figure 3C,D). In conclusion, our findings revealed that GLS was critical for PCa cell proliferation.

### GLS knockdown induces apoptosis and cell cycle arrest of PCa cells

**Figure 4:**
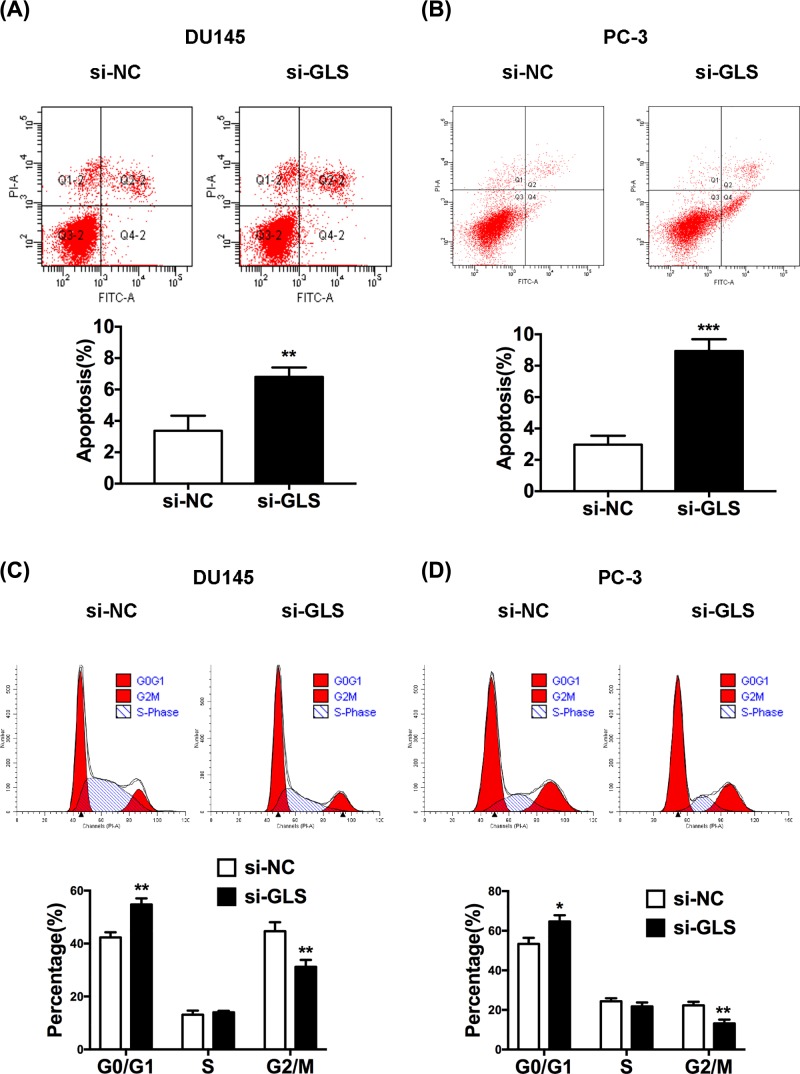
Knockdown of GLS induced apoptosis and cell cycle arrest in PCa cells (**A,B**) Apoptosis percentage of transfected DU145 and PC-3 cells were analyzed by flow cytometry. (**C,D**) Cell cycle distribution of transfected DU145 and PC-3 cells were analyzed by flow cytometry. *P<0.05, **P<0.01, ***P<0.001.

Decreased cell proliferation induced by GLS knockdown may be a consequence of increased cell death. We thus determined whether apoptosis participated in GLS-regulated proliferation of PCa cells using flow cytometry assay. Results indicated that knockdown of GLS remarkably promoted apoptosis in PCa cells (Figure 4A,B). Because cell cycle is the primary event of cell proliferation, we focused on whether GLS regulated the progression of cell cycle. Our results showed that the percentage of cells was significantly increased in G_0_/G_1_ phase while decreased in G_2_/M phase when GLS expression was inhibited (Figure 4C,D), indicating that down-regulation of GLS expression could arrest more cells at G_0_/G_1_ phase, reduce the ratio of cells at S and G_2_/M phases, and thus, inhibite the proliferation of PCa cells. Our findings revealed that knockdown of GLS may inhibit proliferation by inducing apoptosis and cell cycle arrest in PCa cells.

### GLS knockdown alters multiple apoptosis and cell cycle related protein levels in PCa cells

**Figure 5:**
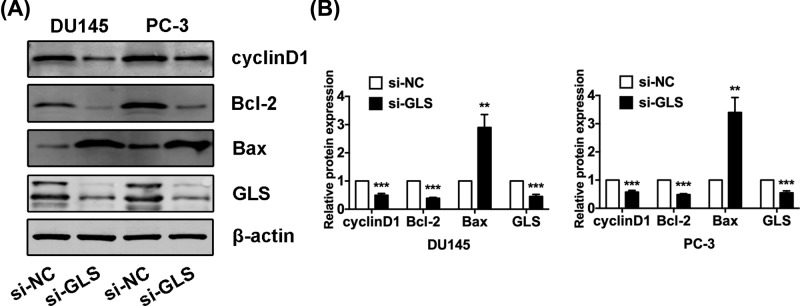
Knockdown of GLS altered multiple apoptosis and cell cycle related protein levels in PCa cells (**A**) The protein bands of cylclinD1, Bcl-2, Bax and GLS were detected by Western blot in both DU145 and PC-3 cells. (**B**) The protein expression of cyclinD1, Bcl-2, Bax and GLS by Western blot analysis. **P<0.01, ***P<0.001.

To further understand the molecular changes involved in GLS-mediated proliferation, apoptosis and cell cycle distribution, we analyzed several apoptosis and cell cycle related protein expressions in PCa cells after GLS knockdown. Bcl-2 and Bax play key roles in apoptosis regulation, where Bcl-2 promotes anti-apoptotic and Bax has an apoptotic function. To investigate the underlying mechanism of the growth inhibitory effects of GLS knockdown, cell cycle regulators cyclinD1 critical to the G_0_/G_1_ phase checkpoint was evaluated. Our data showed that the expressions of Bax were increased while the expressions of cyclinD1 and Bcl-2 were decreased after transfected with si-GLS in PCa cells (Figure 5). Collectively, these data indicated that the GLS may exert a significant inhibitory effect on proliferation by regulating multiple apoptosis and cell cycle related protein levels in PCa.

### GLS knockdown suppresses the Wnt/β-catenin pathway

**Figure 6:**
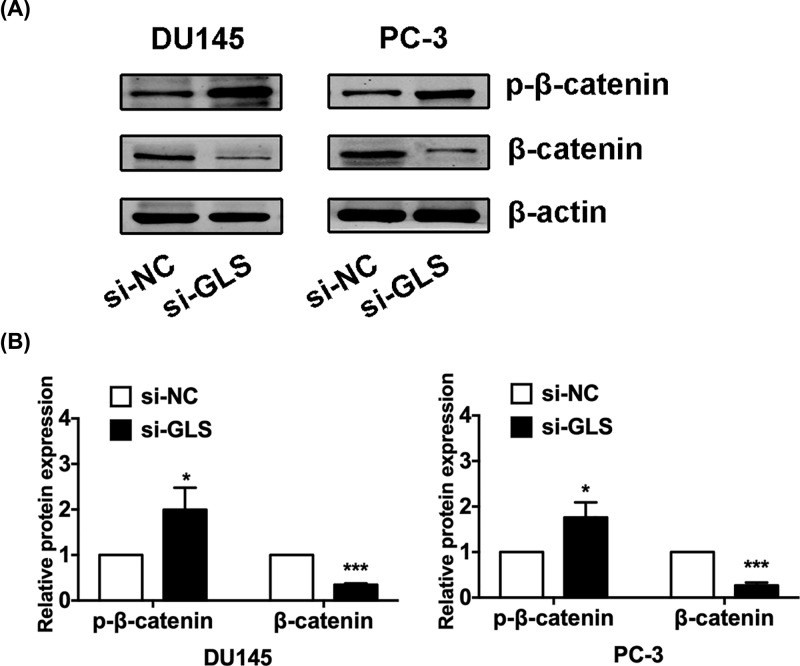
Knockdown of GLS suppressed the Wnt/β-catenin pathway in PCa cells (**A**) The protein bands of p-β-catenin, β-catenin were detected by Western blot in both DU145 and PC-3 cells. (**B**) The protein expression of p-β-catenin and β-catenin by Western blot analysis. *P<0.05, ***P<0.001.

GLS knockdown reduced the expressions of cyclinD1 and Bcl-2, both of which are Wnt/β-catenin pathway regulated molecules. Since Wnt/β-catenin signaling pathway plays a key role in the regulation of tumor progression and is aberrantly activated in PCa, we then examined the role of GLS in Wnt/β-catenin signaling. Western blot analysis revealed that silencing of GLS reduced nuclear β-catenin expression (Figure 6), indicating that down-regulation of GLS expression could inhibite the Wnt/β-catenin signaling. These data together suggested that GLS accelerated cancer progression might be at least in part via up-regulation of β-catenin in PCa.

## Discussion

PCa is the most commonly diagnosed malignancy in men and a major cause of cancer deaths [1]. Besides active surveillance, the primary treatment options for patients with PCa include radical prostatectomy, radiotherapy, hormonal therapy, or combinational therapy [16]. The lack of efficacious therapeutics for advanced PCa has necessitated the development of novel therapeutic agents. Much progress had been made in research for PCa in the past decade [17]. However, the molecular mechanism of its pathogenesis is still poorly understood. Thus, understanding of PCa genetics and molecular pathogenesis will be key to improvements in and personalization of the management of PCa.

GLS is a crucial enzyme in glutamine metabolism as it catalyzes the transformation of glutamine to glutamate, which is further converted into produce α-ketoglutarate [18]. It plays a vital role in up-regulating cell metabolism for tumor growth and is considered to be a potential therapeutic target for cancer treatment [19,20]. GLS has been widely reported to be overexpressed in pancreas [8], bladder [9], lung [10], and breast cancers [11]. Xia et al. [21] reported that the GLS mRNA was significantly up-regulated in neuroblastoma tissues compared with their adjacent normal tissues. Cassago et al. [22] showed that elevated GLS expression was associated with high grade and metastatic breast cancer. In addition, Kim et al. [23] reported that GLS expression in tumor cells was significantly associated with poor disease-free survival in breast cancer patients. With regard to PCa, Pan et al. [14] reported that GLS promoted glucose utilization via glutaminolysis and was highly correlated with the progression in PCa patients. In the present study, we found that GLS is overexpressed in PCa tissues and cell lines. These results agree with our previous study of GLS in bladder cancer (J.Z., unpublished data). We explored the potential correlation between GLS mRNA expression and the various clinicopathological characteristics of PCa patients, and found that the increased expression of GLS was non-significant in terms of Age, PSA level, Lymphatic invasion, and Seminal vesicle invasion. However, we revealed that GLS up-regulation was associated with Gleason score (*P*=0.001) and Tumor stage (*P*=0.015) (Table 1). Collectively, these data suggested that GLS may be implicated in the development and progression of PCa.

To further elucidate its role in the progression of PCa, we explored the biological effects of GLS on PCa cells proliferation, apoptosis, and cell cycle distribution. We demonstrated that knockdown of GLS significantly inhibited the proliferation and induced apoptosis and cell cycle arrest in DU145 and PC-3 cells. Our results suggest that GLS is up-regulated and may act as an oncogene in PCa. These data are consistent with previous studies, which suggest suppression of GLS has antitumor activity across a variety of tumor types, including lymphoma, glioma, breast, pancreatic, lung, and renal cancers [24, 25].

Luan et al. [7] documented that GLS knockdown significantly repressed the glutamine catabolism and growth of melanoma cells. Li et al. [26] demonstrated a promising synthetic lethality strategy by targeting Hsp90 and GLS *in vitro* and in a xenograft tumor model. Masamha et al. [27] reported that inhibition of GLS-sensitized drug-resistant ovarian cancer cells to chemotherapy. We detected several apoptosis and cell cycle related proteins expression in PCa cells after GLS knockdown. We observed that the expressions of Bax were increased while the expressions of cyclinD1 and Bcl-2 were decreased after knockdown of GLS in PCa cells. These suggest that GLS may affect cell proliferation, apoptosis and cell cycle distribution by regulating the levels of Bax, cyclinD1, and Bcl-2 in PCa. Since both cyclinD1 and Bcl-2 are Wnt/β-catenin pathway regulated molecules. In addition, the functions of Wnt/β-catenin signaling in PCa development and progression have been well documented. Therefore, we then examined the role of GLS in Wnt/β-catenin signaling pathway. We found that silencing of GLS induced suppressing of Wnt/β-catenin signaling via directly down-regulating nuclear β-catenin. In this respect, further investigation of the mechanisms by which β-catenin protein is reduced in PCa with GLS knockdown will eventually lead to the development of a new therapeutic strategy for the treatment of PCa.

In summary, we demonstrated that GLS functions as an oncogene in PCa. GLS is overexpressed in PCa tissues and cell lines, and knockdown of GLS significantly inhibited proliferation and induced apoptosis and cell cycle arrest in PCa cells. Additionally, GLS may exert its functions by regulating the expression of Bax, cyclinD1, and Bcl-2 in PCa. Moreover, we found that GLS knockdown suppressed the Wnt/β-catenin pathway. Though these preliminary data show the potential role of GLS in PCa, the underlying molecular mechanism of this process still need to be studied further.

## Supporting information

**Supplementary Figure S1:** 

## Abbreviations

CCK-8: cell counting kit-8
GLS: glutaminase
PCa: prostate cancer
PI: propidium iodide
qRT-PCR: quantitative real-time PCR
