# Supplementary figures and images for: Inhibition of GLS suppresses proliferation and promotes apoptosis in prostate cancer
Source: Biosci Rep. 2019 Jun 25;39(6):BSR20181826. doi: 10.1042/BSR20181826 (PMC6591571; doi:10.1042/BSR20181826)

# RWPE-1

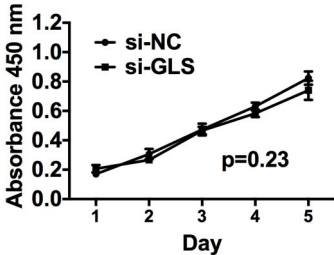

Supplement: Supplementary file 1 [file bsr20181826_Supp1.pdf]
